# Geographic patterns and determinants of antibiotic resistomes in coastal sediments across complex ecological gradients

**DOI:** 10.3389/fmicb.2022.922580

**Published:** 2022-11-03

**Authors:** Shangling Xiong, Kai Wang, Huizhen Yan, Dandi Hou, Yanting Wang, Meng Li, Demin Zhang

**Affiliations:** ^1^State Key Laboratory for Managing Biotic and Chemical Threats to the Quality and Safety of Agro-products, School of Marine Sciences, Ningbo University, Ningbo, China; ^2^College of Biological and Environmental Sciences, Zhejiang Wanli University, Ningbo, China; ^3^Key Laboratory of Marine Biotechnology of Zhejiang Province, Ningbo University, Ningbo, China; ^4^Collaborative Innovation Center for Zhejiang Marine High-Efficiency and Healthy Aquaculture, Ningbo, China

**Keywords:** antibiotic resistance genes, geographic patterns, coastal sediment, terrestrial runoffs, mariculture

## Abstract

Coastal areas are highly influenced by terrestrial runoffs and anthropogenic disturbances, commonly leading to ecological gradients from bay, nearshore, to offshore areas. Although the occurrence and distribution of sediment antibiotic resistome are explored in various coastal environments, little information is available regarding geographic patterns and determinants of coastal sediment antibiotic resistomes across ecological gradients at the regional scale. Here, using high-throughput quantitative PCR, we investigated the geographic patterns of 285 antibiotic resistance genes (ARGs) in coastal sediments across a  ~  200  km scale in the East China Sea. Sediment bacterial communities and physicochemical properties were characterized to identify the determinants of sediments antibiotic resistome. Higher richness and abundance of ARGs were detected in the bay samples compared with those in nearshore and offshore samples, and significant negative correlations between the richness and/or abundance of ARGs and the distance to coastline (DTC) were identified, whereas different types of ARGs showed inconsistency in their relationships with DTC. The composition of antibiotic resistome showed significant correlations with nutrition-related variables (including NH_4_^+^-N, NO_3_^−^-N, and total phosphorus) and metals/metalloid (including As, Cu, Ni, and Zn), suggesting that terrestrial disturbances largely shape the antibiotic resistome. The Bipartite network showed strong associations between ARGs and mobile genetic elements (MGEs), and Partial Least Squares Path Modeling further revealed that terrestrial disturbance strength (as indicated by DTC) directly affected abiotic environmental conditions and bacterial community composition, and indirectly affected antibiotic resistome *via* MGEs. These findings provide insights into regional variability of sediment antibiotic resistome and its shaping path across complex ecological gradients, highlighting terrestrial disturbances as determinative forces in shaping coastal sediment antibiotic resistomes.

## Introduction

Antibiotic resistance has become one of the major threats to the health of humans and animals in 21^st^ century (Berendonk et al., 2015). A considerable body of evidence has confirmed that the propagation of antibiotic resistance is mainly due to the proliferation of antibiotic resistance genes (ARGs), which can be frequently spread among different microbial species *via* horizontal transfer (Willems et al., 2011; Stecher et al., 2013). As a result, ARGs have been unexpectedly detected in diverse environments, such as drinking water (Shi et al., 2013), estuarine sediment (Zhu et al., 2017), deep ocean sediment (Chen et al., 2013b), and even Antarctic soil (Wang F. et al., 2016). The growing spread of ARGs without effective restrictive approaches will shape the antibiotic resistome of earth’s microbiome, thus threatening the health of our planet (Zhu and Penuelas, 2020).

Population density in coastal regions is approximately three times higher than the global average (Small and Nicholls, 2003). Coastal area is the transitional zone in the transportation of anthropogenic contaminants from terrestrial sources to the ocean (Wu et al., 2019). ARGs in high abundance have been detected in coastal sediments across various regions (Zhu et al., 2017; Griffin et al., 2019; Zhao et al., 2020). A few studies reported maricultural farms as a point-source (Lin et al., 2015; Muziasari et al., 2016; Gao et al., 2018) and terrestrial runoffs as a primary non-point source (Chen et al., 2013a,b) leading to the enrichment of ARGs in coastal sediments. Terrestrial runoffs commonly lead to multiple environmental gradients from bay to offshore areas (Hou et al., 2014; Wang et al., 2015; Zhang H. et al., 2018; Broman et al., 2022). However, our knowledge about geographic patterns of antibiotic resistomes in sediments across coastal ecological gradients is still lacking.

The antibiotic resistome of coastal sediments is likely governed by the interplay of multiple ecological determinants. Emerging studies have demonstrated that pollutants (such as heavy metals and antibiotic residues; Chen Y. et al., 2020) and physicochemical properties of sediment (such as water content, texture, salinity of pore water, total carbon, and total nitrogen; Zhang et al., 2019, 2020) were both important on shaping antibiotic resistome. Moreover, physicochemical properties of sediment can impose selective pressure on microbial community composition (Wang K. et al., 2016), which may subsequently drive the spread of ARGs. However, little is known about the relative importance and shaping path of key determinants of antibiotic resistomes in coastal sediments across multiple environment gradients.

The coastal area of northern Zhejiang Province, China, is located at the downstream of the Yangtze River Estuary and Qiantang River Estuary, East China Sea, adjacent to several mega/big cities including Shanghai, Hangzhou, and Ningbo with intensive urbanization and industrialization. Among the representative zones of this area, Hangzhou Bay is one of the largest bays in China, where the Qiantang River (the largest river in Zhejiang) enters the sea. Xiangshan Bay and Sanmen Bay are typical semi-enclosed with extensive aquaculture activities and weak water-exchange ability. Yushan Islands Reservation (a national marine ecosystem reserve area) and the Eastern Boundary of the Island-chain serve as the control zones with minimal terrestrial disturbance. This coastal area receives a tremendous amount of river runoffs wrapping around terrestrial pollutants from industrial and agricultural discharges as well as and urban effluents, which are partially transferred and accumulated into the sediments. Previous studies have reported multiple environment gradients, including salinity and nutrients (Wang et al., 2015), heavy metals (Jiang et al., 2018), and polychlorinated biphenyls (Zhao et al., 2019), from the bays to offshore, as well as high abundances of ARGs in some of estuarine sediment samples there (Zhu et al., 2017; Chen et al., 2019). Nevertheless, how and to what extent the ARG profiles of sediments vary with ecological gradients have not been comprehensively investigated. This coastal area provides an ideal model to clarify the regional variability of antibiotic resistome across coastal multiple environmental gradients.

In this study, we collected surface sediment samples from seven representative zones of coastal area of northern Zhejiang across a ~ 200 km scale. Using high-throughput quantitative PCR (HT-qPCR) with 285 validated primer sets targeting nine types of ARGs, we uncovered the comprehensive profiles of sediment antibiotic resistome. By integrating the quantification of antibiotics, environmental variables, mobile genetic elements (MGEs) and 16S rRNA gene amplicon sequencing, we aim to (1) characterize geographic patterns of sediment antibiotic resistomes across complex ecological gradients; (2) explore the relative importance and interplay of biotic factors (bacterial community and MGEs) and abiotic environmental factors (antibiotics and physicochemical properties) in shaping sediment antibiotic resistomes.

## Materials and methods

### Study area, sample collection, and measurements of sediment physicochemical properties

**Figure 1:**
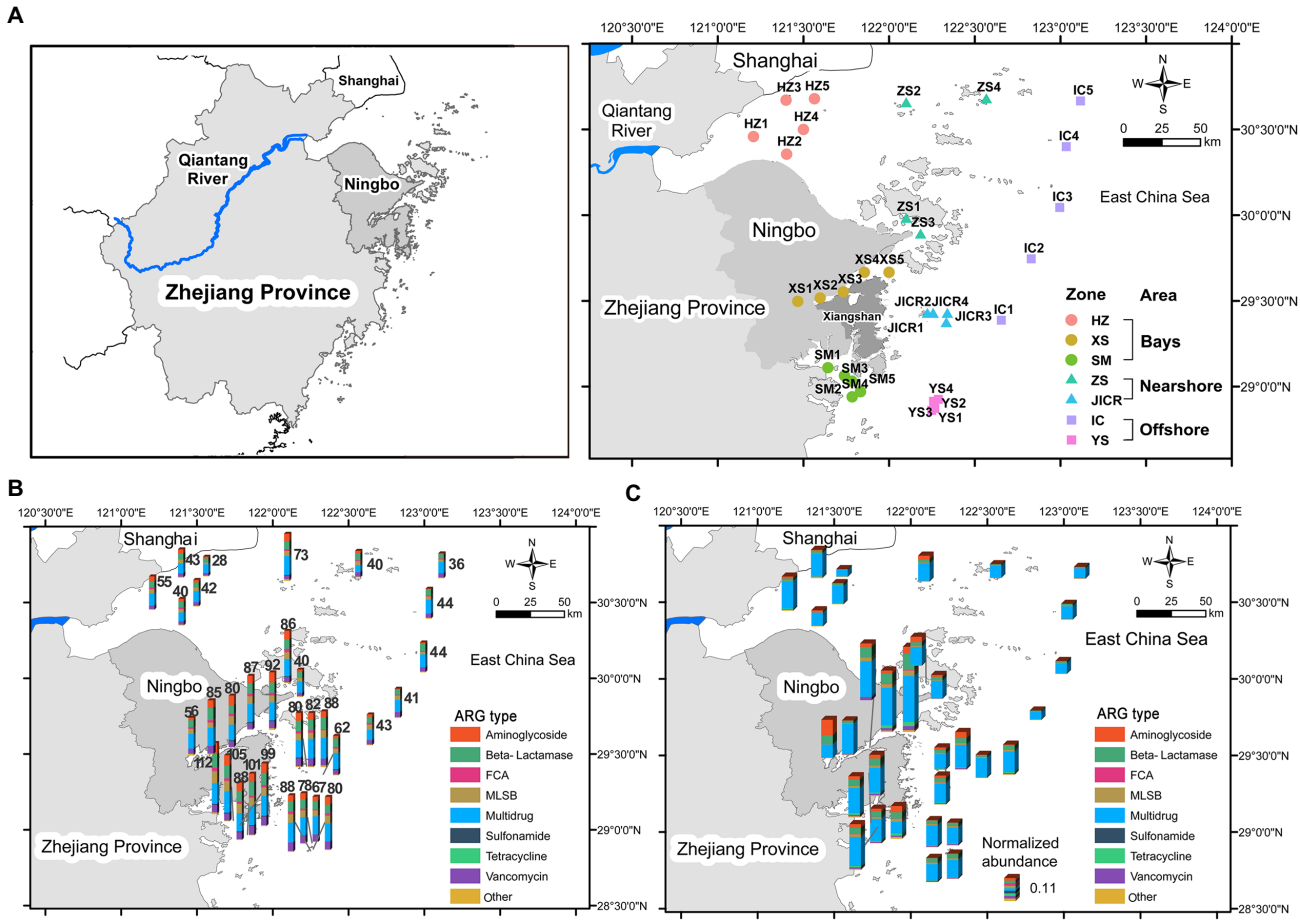
The map of sampling stations across the coastal northern Zhejiang, in the East China Sea **(A)**. Histogram maps showing the spatial distribution of richness **(B)** and normalized abundance **(C)** of antibiotic resistance genes (ARGs) across the study region. ARGs were classified based on the type of antibiotics that they confer resistance to. HZ, Hangzhou Bay; XS, Xiangshan Bay; SM, Sanmen Bay; ZS, Zhoushan Islands; JICR, Jiushan Islands Conservation and Reservation; IC, the Eastern Boundary of the Island-Chain; YS, Yushan Islands Reservation.

During a summer cruise (August 5–20, 2015), we collected 32 surface sediment samples from 32 stations across seven representative zones of coastal northern Zhejiang, including Hangzhou Bay (HZ), Xiangshan Bay (XS), and Sanmen Bay (SM) as bay areas, Zhoushan Islands (ZS) and Jiushan Islands Conservation and Reservation (JICR) as nearshore areas, and Yushan Islands Reservation (YS) and the Eastern Boundary of the Island-chain (IC) as offshore areas (Figure 1A). Surface sediment samples (top 2 cm) were collected using a custom-made corer and were mixed well in a sterile glass bottle. The sample were partly transferred to sterile cryovials and were immediately stored in liquid nitrogen prior to DNA extraction. The fresh subsamples were stored at 4°C prior to analyses. Sediment texture (expressed as proportions of clay, silt, and sand) was measured using laser grain-size analysis (Bettersize 2000LD, China). Total carbon (TC), total nitrogen (TN), and total sulfur (TS) in the sediments were determined using an Elementar Vario EL II analyzer (Elementar Analysensysteme GmbH, Germany). Five grams of dry sediment mixed well with 12.5 ml Ultrapure water was subjected to pH measurement with a SenTix meter (WTW, Germany). NO_3_^−^-N and NH_4_^+^-N in the sediments were extracted with 2 M KCl solution at 25°C, and the concertation of NO_3_^−^-N and NH_4_^+^-N in extracts was determined using a continuous flow autoanalyzer (B&L, Germany). Total phosphorus (TP) in extracts was determined with molybdenum blue spectrophotometry. Heavy metals/metalloid (including arsenic (As), cadmium (Cd), chromium (Cr), copper (Cu), mercury (Hg), nickel (Ni), lead (Pb), and zinc (Zn)) in the sediments were measured as previously described (Yuan et al., 2004), with modifications in the digestion reagent, comprising nitric acid, perchloric acid, and hydrofluoric acid (5:1:1, *v/v/v*). Subsequently, the concentrations of metals in the extracts were determined using inductively coupled plasma mass spectrometry (Agilent 7500A, United States). The concentrations of As and Hg were determined using the atomic fluorescence spectrometer (Rayleigh AF-640A, China).

### Measurement of antibiotics in sediment

To understand the overall level of antibiotics in the sediments, the concentrations of 27 target antibiotics were measured. They belong to six major antibacterial classes: tetracyclines (including tetracycline, oxtetracycline, methacycline, chlortetracycline, and doxycycline); sulfonamides (including sulfadiazine, sulfamethoxazole, sulfamethazine, sulfamonomethoxine sodium hydrate, sulfachinoxalin, sulfadimethoxine, sulfameter, sulfaclozine sodium monohydrate, sulfathiazole, and sulfamerazine); diaminopyrimidines (trimethoprim); fluoroquinolones (including fleroxacin, ofloxacin, enrofloxacin, marbofloxacin, pefloxacin, difluoxacin, and lomefloxacin); macrolides (including erythromycin, clarithromycin, roxithromycin, and tylosin); and chloramphenicols (chloramphenicol). Procedures of the sample pretreatment and detection for antibiotics were previously described (Zhou et al., 2012). Briefly, the sediment samples were successively lyophilized, powdered, and passed through a 50-mesh sieve, and then the antibiotics in the sediments were ultrasonically extracted with acetonitrile for 12 min. The extracted supernatants were passed through the strong anion exchanger cartridges (Thermo, United States) and then hydrophilic–lipophilic balance cartridges (Waters, United States) for cleaning up and enrichment. The final eluates were evaporated to dryness using a nitrogen blower, dissolved with acetonitrile to 2 ml, filtered through a 0.2-μm nylon membrane, and then analyzed using high performance liquid chromatography–tandem mass spectrometry (Waters, United States). Solvent and procedural blanks were applied to ensure the accuracy of the quantification. The recovery rates of 27 antibiotics in the spiked samples range 48.7–95.1% with 88.7% as the median.

### DNA extraction, 16S rRNA gene sequencing, and sequence processing

The DNA from sediment samples (0.5 g dry-weight basis) was extracted using a Power Soil DNA Isolation Kit (MOBIO, United States) and then stored at -80°C. To characterize bacterial community composition, the V4 region of 16S rRNA gene was amplified with the primers: 515FY (5′- GTGYCAGCMGCCGCGGTAA-3′) and 806RB (5′- GGACTACNVGGGTWTCTAAT-3′) with dual barcodes (Apprill et al., 2015; Walters et al., 2016). The PCR was performed with a 20-μL reaction system under the following conditions: initial denaturation at 95°C for 3 min, and then 27 cycles of the following program were used for amplification: 95°C for 30 s, 55°C for 30 s and 72°C for 45 s, plus a final extension of 10 min at 72°C. Triplicate PCR products for each sample were pooled and purified using a PCR fragment purification kit (TaKaRa, Japan). The purified PCR products were assayed for fragment size with an Agilent 2100 Bioanalyzer (Agilent, United States) and quantified using a Quant-It Pico Green kit (Invitrogen, United States) and Qubit fluorometer (Life Technologies, United States). Sequencing was performed using the Illumina MiSeq platform (Illumina, United States). All sequences were deposited in the NCBI SRA under the accession number SRP156486.

Raw FASTQ files were de-multiplexed with QIIME v1.9.1 (Caporaso et al., 2010), and FLASH was used to joined the paired reads (Magoč and Salzberg, 2011). The sequences were quality filtered at Q20 using the script *split_libraries_fastq.py* with QIIME v1.9.1 (Bokulich et al., 2013). The remaining sequences were chimera detected using UCHIME (Edgar, 2010). After removing the chimeras, the filtered reads were clustered into operational taxonomic units (OTUs) with 99% sequence similarity using the *pick_open_reference_otus.py* script with the Sortmerna_sumclust method (Kopylova et al., 2012). The representative sequence of each OTU was taxonomically assigned against the SILVA 132 database.^1^ Archaea, chloroplast, mitochondria, singletons, and the other OTUs unassigned to bacteria were removed. A total of 914,460 qualified bacterial sequences and 47,788 OTUs were generated from all samples. The bacterial OTU table was rarefied at 21,300 reads per sample prior to downstream analyses.

### Quantification of ARGs and MGEs

High-throughput qPCR reactions were performed to evaluate the relative abundance of ARGs using a SmartChip Real-time PCR system (Wafergen Inc., United States) as previously described (Su et al., 2015). A total of 296 primer sets, targeting 285 ARGs, 10 MGEs (including 8 transposase genes, a class I integron-integrase gene, and a clinic integron-integrase gene), and 16S rRNA gene, were used. Detailed description of experimental procedures and data analyses can be found in previous studies (Su et al., 2015; Liu et al., 2018). The normalized abundance of ARGs and MGEs were calculated as previously described (Ouyang et al., 2015). Each sample was tested with three technical replicates, and DNA-free water was used as the negative control. An amplification efficiency of 2 ± 0.2 and unique peaks were set for data validation. A threshold cycle (*C_t_*) value of 31 was the detection limit. The relative copy number of ARGs, MGEs, and 16S rRNA gene was calculated based on *C_t_* values using the following equation (Looft et al., 2012; Zhu et al., 2018):


(1) $$\mathrm{Relative}\;\;\mathrm{Gene}\;\;\mathrm{Copy}\;\mathrm{Number}=10^{\left(\left(31-Ct\right)/\left(10/3\right)\right)}$$

The normalized copy number of ARGs and MGEs was calculated as following (Zhu et al., 2018):


(2) $$\begin{array}{l} \mathrm{Normalized}\;\mathrm{ARG or MGE}\;\mathrm{Copy Number}\\ =\left(\begin{array}{l} \mathrm{Relative}\;\mathrm{ARG or MGE}\;\;\mathrm{Copy Number}/\\ \mathrm{Relative}\;16S\;\mathrm{rRNA}\;\mathrm{Gene Copy Number} \end{array}\right)\times 4.1 \end{array}$$

### Statistical analyses

The map of sampling stations, spatial visualization of ARGs, MGEs, environmental variables, antibiotics, and the calculation of the distance of each station to the coastline (DTC) were performed using ArcGIS 10.4. Pearson correlations between environmental variables and DTC were tested using the “lm ()” function of the R package “vegan” (Oksanen et al., 2020). Spearman’s correlations between different types of antibiotics and ARGs were tested and visualized using the R package “corrplot” (Wei and Simko, 2021). The significance of differences in ARG richness and normalized abundance of ARGs and MGEs among bay, nearshore, and offshore areas was tested by Wilcoxon rank-sum tests using the R package “stats” (R Core Team, 2020). The linear regression model of the richness and abundance of ARGs and the abundance of MGEs with DTC were evaluated using the “lm ()” function of the R package “vegan.” Principal Coordinate Analysis (PCoA) based on Bray–Curtis dissimilarity was applied to visualize the compositional variation of antibiotic resistomes and bacterial communities. To evaluate the significance of difference in the composition of ARGs between zones, Permutational Multivariate Analysis of Variance (PERMANOVA) and Analysis of Similarity (ANOSIM) were performed using the R package “vegan.” Box plots were used to show the compositional dissimilarity of antibiotic resistomes among stations in each zone. “Indicator ARGs” of each zone was identified using the R package “indicspecies” (Cáceres and Legendre, 2009). The distance-decay model was fitted with Bray–Curtis similarity of antibiotic resistomes and geographic distance between stations. The correlation between antibiotic resistome and bacterial community (as biotic variables) was tested using Procrustes analysis and Mantel test (Oksanen et al., 2020). Bipartite network analysis was applied to show co-occurrence patterns of ARGs and MGEs detected in at least 50% of samples using CoNet (Soffer et al., 2015; Faust and Raes, 2016). To ensure the robustness of associations, the edges between ARGs and MGEs were tested by calculating Spearman correlation, Pearson correlation, Kullback–Leibler dissimilarity, and Bray–Curtis dissimilarity. The edges with correlation coefficient > 0.8 and *p* < 0.01 by at least two methods were considered statistically robust as suggested previously (Soffer et al., 2015) and then were visualized in the final network using Gephi (Bastian et al., 2009). Redundancy analysis (RDA) was used to co-plot environmental variables with clusters of antibiotic resistomes using the R package “vegan,” with the “vif.cca” function to pre-filter redundant variables (variance inflation factors >10) to avoid the collinearity issues and the “envfit” function to select variables (*p* < 0.05) for generating the final ordinations. To evaluate the direct and indirect influence of potential determinants (including five categories: DTC, antibiotics, abiotic environmental variables, bacterial community, and MGEs) on the compositional variation of antibiotic resistomes, Partial Least Squares Path Modeling (PLS-PM) was constructed using the R package “plspm” (Wetzels et al., 2009). The goodness-of fit index (*GoF*) was used to evaluate overall prediction performance of the model, and higher values indicate better prediction performance.

## Results

### Regional variability of the concentration and composition of antibiotics

Twelve target antibiotics affiliated to five antibacterial classes (except diaminopyrimidines) were detected in at least one sample (Supplementary Figure S1). Macrolides and fluoroquinolones were detected in all samples. Tetracyclines, chloramphenicols, and sulfonamides were detected in 40.6, 31.2, and 6.3% of samples, respectively. The average concentration of different classes of antibiotics followed the order as: tetracyclines > fluoroquinolones > macrolides > chloramphenicols > sulfonamides. The concentration of total target antibiotics in each sample was highly variable, ranging from 2.48 to 149.01 μg kg^−1^ (34.59 μg kg^−1^ in average). For individual antibiotics, marbofloxacin was detected in 90.6% of all samples, followed by roxithromycin (81.3%) and pefloxacin (71.9%). Six fluoroquinolone antibiotics were detected at concentrations ranging from 2.19 to 12.50 μg kg^−1^. Likewise, three macrolide antibiotics were detected at concentrations ranging from 1.96 to 9.77 μg kg^−1^. Methacycline was the one with the highest average concentration up to 13.80 μg kg^−1^. In general, the average concentration of total target antibiotics in nearshore samples (50.08 μg kg^−1^) were higher than that in bay (29.74 μg kg^−1^) or offshore samples (18.91 μg kg^−1^; Supplementary Figure S1).

### Geographic patterns of environmental variables

Across a ~ 200 km scale, nutrient-related variables like ammonium (NH_4_^+^-N, 1.9–23.4 mg kg^−1^), nitrate (NO_3_^−^-N, 2.6–20.4 mg kg^−1^), and total phosphorus (TP, 667–1,627 mg kg^−1^) gradually decreased from bay to offshore areas, and metals/metalloid including As, Cu, Ni, Pb, and Zn generally exhibit a similar pattern (Supplementary Figure S2). Furthermore, nutrient-related variables (including NH_4_^+^-N, NO_3_^−^-N, and TP) and metals/metalloid (including As, Cu, Ni, and Zn) showed significant negative correlations with distance to coastline (DTC; all |r| ≥ 0.343, Supplementary Table S1), while total carbon (TC) and pH show a reverse gradient with higher levels in the offshore area, confirming the existence of multiple environmental gradients. In natural environments, higher levels of nutrients and heavy metals often indicate greater intensity of anthropogenic disturbance (Zhang H. et al., 2018; Vareda et al., 2019). Given the common correlations of the levels of nutrients and heavy metals with DTC, we use DTC as an indicator of anthropogenic disturbance strength at each station, that is, greater DTC presents weaker disturbance.

### Bacterial community composition

Proteobacteria, Chloroflexi, Bacteroidetes, and Actinobacteria were the dominant bacterial phyla, accounting for 80.2% of bacterial sequences. The relative abundance of Deltaproteobacteria was significantly higher in offshore samples than that in bay or nearshore samples, while the opposite pattern was observed in Chloroflexi and Actinobacteria (Data not shown). The PCoA further revealed significant differences in bacterial community composition among bay, nearshore, and offshore samples (PERMANOVA, *p* < 0.01), and PCoA1 value was significantly correlated with DTC (Supplementary Figure S3).

### Geographic patterns of ARGs and MGEs

Overall, a total of 150 ARGs affiliated to 9 types were detected across the sediments, and the numbers of ARGs detected in each station ranged from 28 to 112, with an average of 68 (Figure 1B). With regard to normalized abundance of ARGs, the multidrug resistance genes predominated in almost all stations, with relative abundance ranging from 34.5 to 90.7%, whereas aminoglycoside resistance genes became dominant ARGs in XS1 (40.0%; Figure 1C).

**Figure 2:**
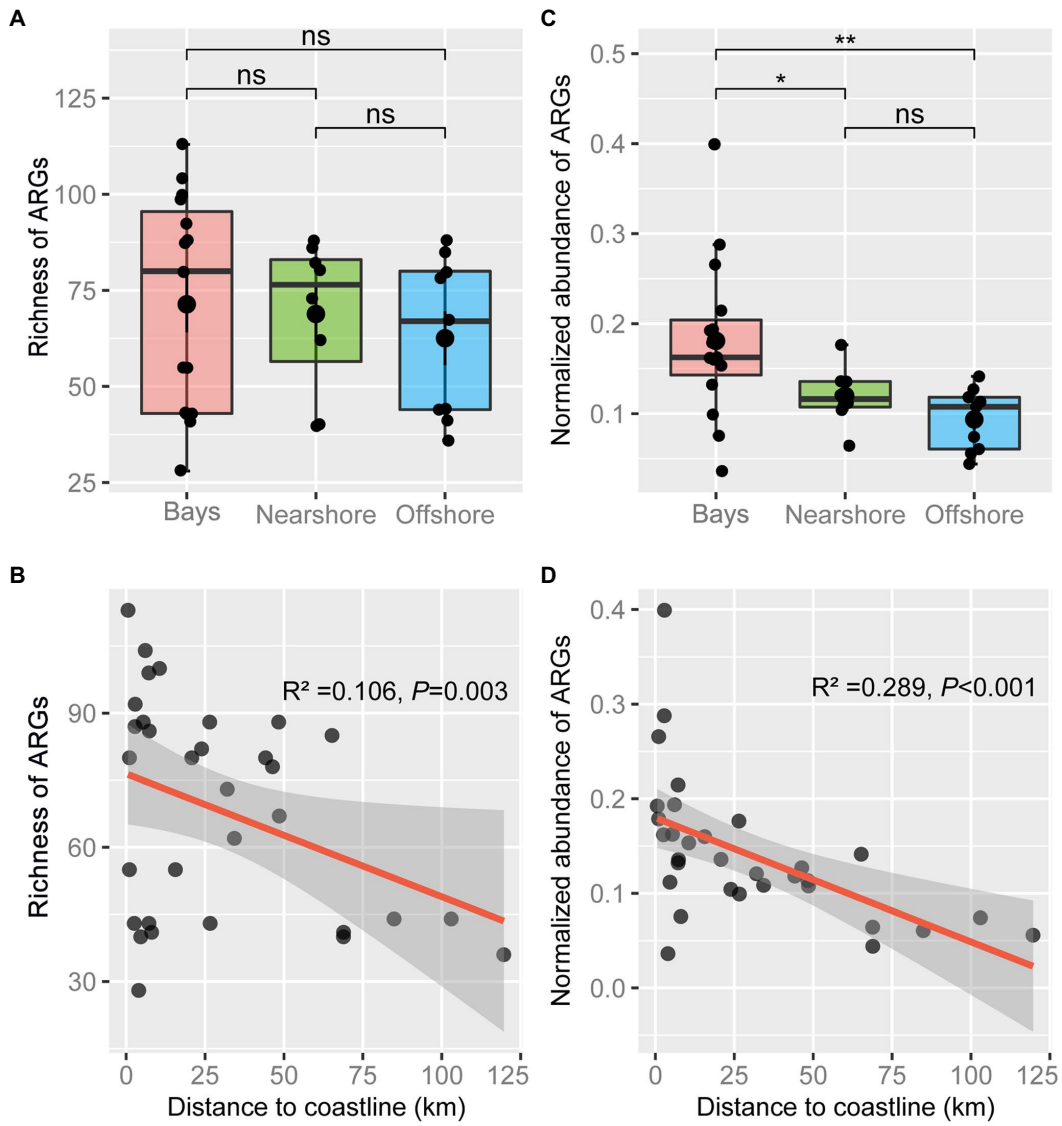
Characteristics of antibiotic resistomes across Bay, Nearshore, and Offshore areas. Boxplots showing the variation in richness **(A)** and normalized abundance **(C)** of antibiotic resistance genes (ARGs). Statistical significance was tested by Wilcoxon rank-sum test (^*^p < 0.05, ^**^p < 0.01, ns: not significant). The lower and upper hinges of the boxes correspond to the 25^th^ and 75^th^ percentiles. The lines in the boxes correspond to median. The upper whisker extends from the hinge to the value no further than 1.5IQR (inter-quartile range). The lower whisker extends from the hinge to the smallest value at most 1.5IQR. Data beyond the end of the whiskers are ‘outlying’ points. Correlations of richness **(B)** and normalized abundance **(D)** of ARGs with distance to coastline (DTC) were shown. The red line represents a linear fit with adjusted R^2^. The shaded area represents 95% confidence intervals.

**Figure 3:**
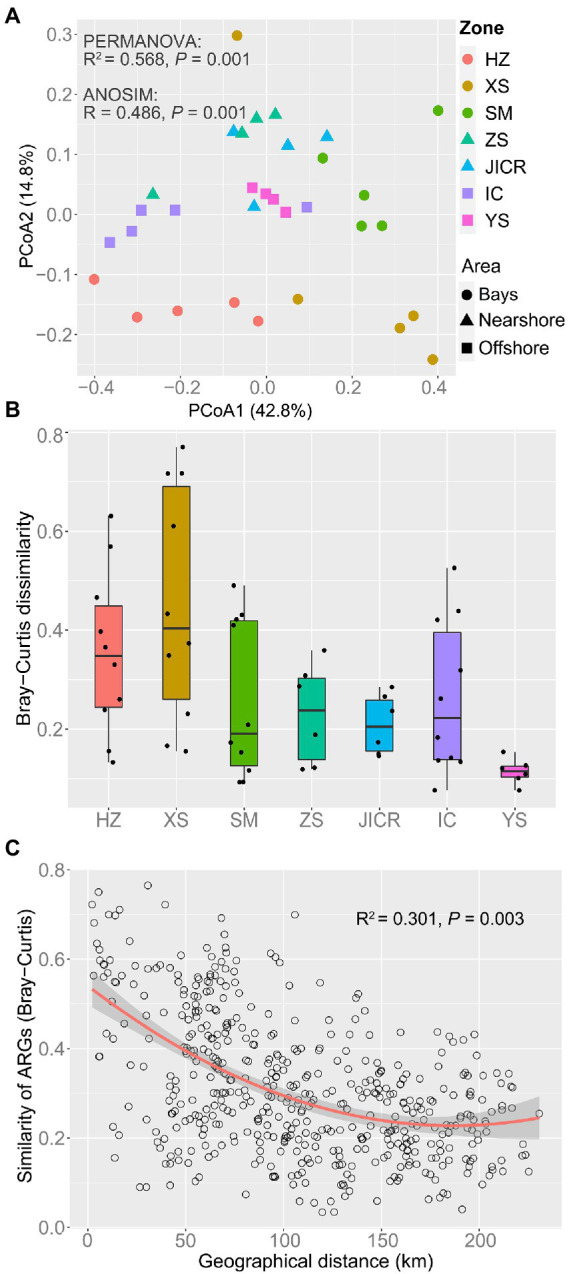
Geographic pattern of antibiotic resistomes across the study region. Principal Coordinate Analysis (PCoA) based on Bray–Curtis dissimilarity visualizing compositional variation of antibiotic resistomes **(A)**. Permutational Multivariate Analysis of Variance (PERMANOVA) and Analysis of Similarity (ANOSIM) were used to test the significance of variation in antibiotic resistomes across zones. Box plots showing the heterogeneity of antibiotic resistomes within each zone **(B)**. Refer to the Figure 2 for the features of the boxes. Distance-decay relationship between Bray–Curtis similarity of antibiotic resistomes and geographic distance between sampling stations **(C)**. The red line represents a binomial fit with adjusted R^2^. The shaded area represents 95% confidence intervals.

Although there was no significant difference in richness of ARGs among bay, nearshore, and offshore samples (Figure 2A), ARG richness showed a significant negative correlation with DTC (*p* = 0.003, Figure 2B). Ninety-five ARGs were shared among the three areas, accounting for 67.9, 77.9, and 94.1% of the detected ARGs in bay, nearshore, and offshore areas, respectively. In addition, 27 ARGs were uniquely detected in bay area, which mainly mediate resistance to aminoglycoside, beta-lactamase, tetracycline, and macrolide-lincosamide-streptogramin B (MLSB) resistance genes. In contrast, only four and one unique ARG(s) was/were detected in nearshore and offshore samples, respectively (Supplementary Table S2). The normalized abundance of total detected ARGs in bay samples was significantly higher than that in nearshore or offshore samples (*p* < 0.05, Figure 2C) and showed a negative correlation with DTC (*p* < 0.001, Figure 2D). Furthermore, the normalized abundances of seven types of ARGs (aminoglycoside-, beta-lactamase-, FCA-, MLSB-, multidrug-, sulfonamide-, and tetracycline resistance genes) showed negative correlations with DTC (all *p* < 0.05), while the abundances of vancomycin resistance genes and others showed non-significant decreasing trend with DTC (Supplementary Figure S4). In general, antibiotic resistomes in the sediments varied with zones, as statistically evidenced by PERMANOVA and ANOSIM (Figure 3A; Supplementary Table S3), and the composition of antibiotic resistomes in HZ were distinct from other zones. Antibiotic resistomes in HZ and XS showed the highest within-zone divergence, while those in JICR and YS showed more homogeneity (Figure 3B). Moreover, between-station similarity of antibiotic resistomes showed a significant distance-decay pattern (R^2^ = 0.301, *p* = 0.003, Figure 3C). Multidrug (*qacEΔ1-01, qacEΔ1-02, acrR-01,* and *qacH-01*) and beta-lactamase (*aadA1* and *aadA-01*) resistance genes were identified as significant indicator ARGs in HZ; tetracycline (*tetT and tetX*), FCA (*floR*), and aminoglycoside (*strB*) resistance genes in XS; MLSB (*ermA*), beta-lactamase (*blaPAO* and *bla-AAC-1*), and sulfonamide (*sul2*) resistance genes in SM; however, no significant indicator ARGs were identified in ZS or JICR (Supplementary Table S4).

Six MGEs, including 4 transposase genes (*tnpA-02, tnpA-04, tnpA-05, and Tp614*), 2 integron-integrase genes (class 1 integron-integrase gene (*intI-1*) and clinical class 1 integron-integrase gene (*cIntI-1*)) were detected across the study region (Supplementary Figure S5). In terms of ubiquity, *tnpA-02* and *tnpA-04* were detected in 87.5% of the stations, while *tnpA-05* and *Tp614* were detected merely in 3 and 4 stations, respectively. The *intI-1* and *cIntI-1* were predominant in all the stations (except for HZ1 and HZ2), with relative abundance in total MGEs ranging from 35.3 to 76.6%, whereas *tnpA-04* became the dominant MGEs in HZ1 (38.8%) and HZ2 (56.2%). There was no significant difference in richness of MGEs among bay, nearshore, and offshore areas (*p* > 0.05, data not shown). The normalized abundance of total detected MGEs in bay samples was significantly higher than nearshore and offshore samples (*p* < 0.05, Supplementary Figure S6A). Additionally, the abundance of MGEs showed a negative correlation with DTC as similar as that of ARGs (R^2^ = 0.167, *p* = 0.026, Supplementary Figure S6B).

### Relationships of antibiotic, environmental variables, bacterial communities, and MGEs with antibiotic resistomes

**Figure 4:**
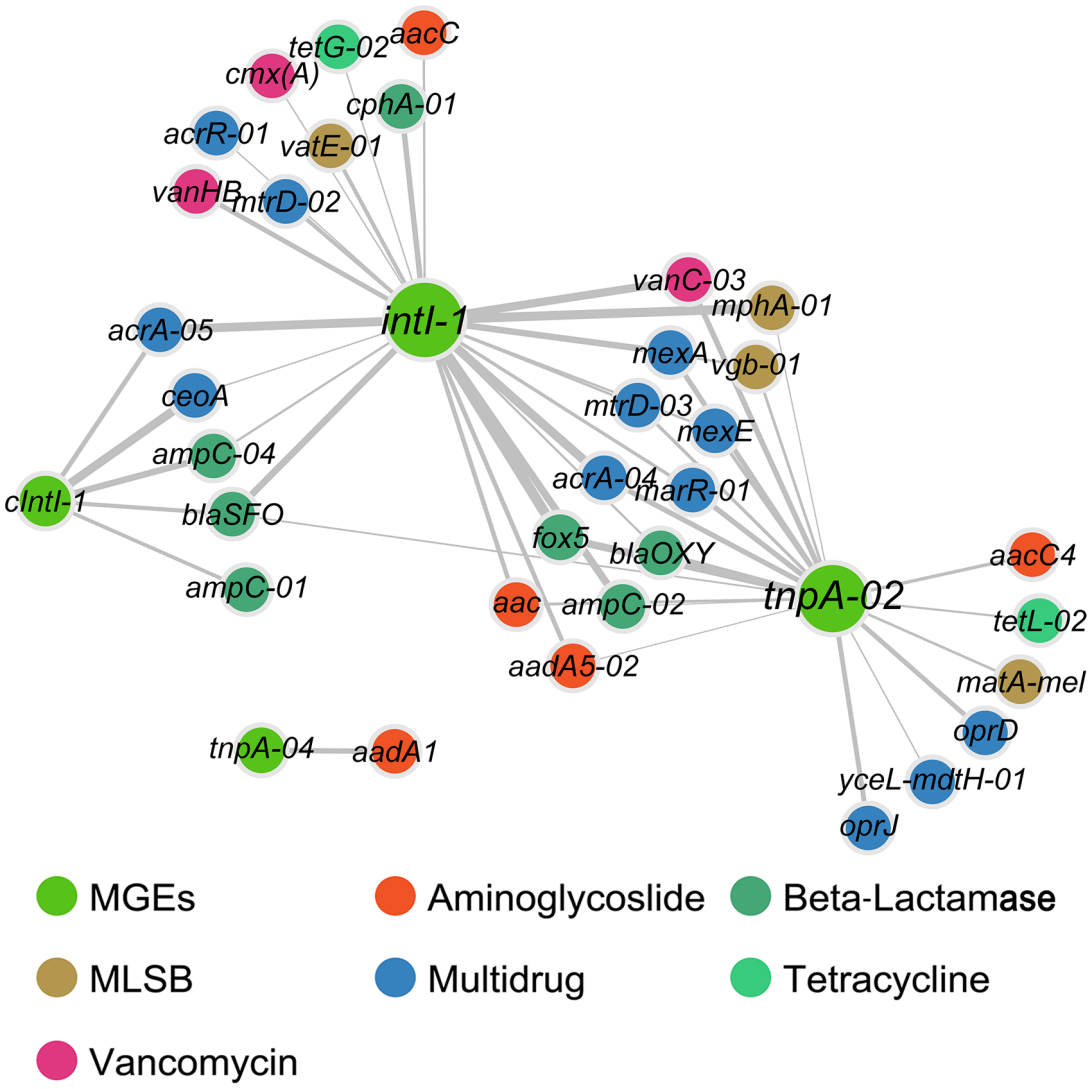
Bipartite network analysis showing associations of MGEs with ARGs. The edges with correlation coefficient > 0.8 and *p* < 0.01 by at least two methods (among Spearman correlation, Pearson correlation, Kullback–Leibler dissimilarity, and Bray–Curtis dissimilarity) were considered statistically robust and visualized. Line thickness is proportional to correlation coefficients.

Environmental variables (including slit, sand, As, and water depth) had strong correlations with the richness and abundance of ARGs, while heavy metals (including Cu, Ni, and Zn) were correlated with the abundance of ARGs (Supplementary Table S1). Furthermore, environmental variables (including TN, NO_3_^−^-N, NH_4_^+^-N, As, Cu, and slit) and DTC were identified as key factors shaping the composition of antibiotic resistomes (all *p* < 0.05, Supplementary Figure S7). No significant correlations were found between the concentrations of antibiotics (either total or individual types) and the abundances of corresponding ARGs (Supplementary Figure S8). Both Procrustes analysis (M^2^ = 0.645, *p* < 0.001) and Mantel test (*r* = 0.379, *p* < 0.001) confirmed the association between bacterial community and antibiotic resistome. The total abundances of ARGs and MGEs were significantly correlated. Moreover, the abundance of integron-integrase genes (*cIntI-1* and *intI-1*) showed significant correlations with all ARG types except aminoglycoside resistance genes, as was the abundance of transposase gene *tnpA-02* (Supplementary Table S5). The network further dissected the relationship between individual ARGs and MGEs, which was composed of 37 nodes (33 ARGs and 4 MGEs) and 51 edges. The *intI-1* gene showed common associations with 25 ARGs. Additionally, the transposase gene *tnpA-02* co-occurred with 19 ARGs, and *tnpA-04* co-occurred with *aadA1* (Figure 4).

**Figure 5:**
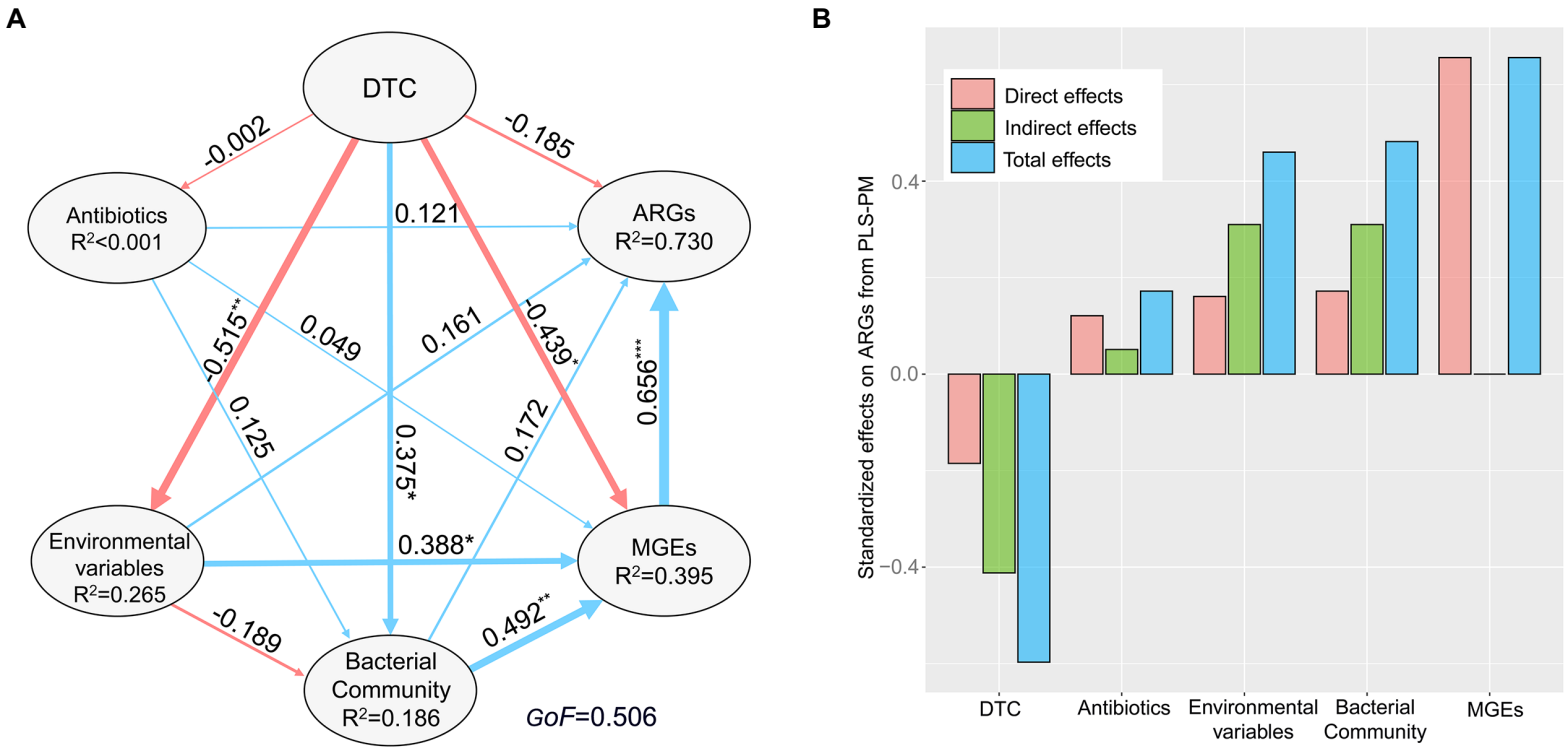
The direct and indirect effects of distance to coastline (DTC), antibiotics, environmental variables, bacterial community, and mobile genetic elements (MGEs) on sediment antibiotic resistomes across the study region. The final path of Partial Least Squares Path Modeling (PLS-PM) **(A)**. The blue and red lines indicate positive and negative direct effects, respectively. ^*^p < 0.05, ^**^p < 0.01, ^***^p < 0.001. Data near each arrow is partial path coefficient (λ) associated with each relationship, and line thickness is proportional to the correlation coefficients. *GoF*, the goodness of fit index of the model. R^2^ values represent the proportion of the variance explained for endogenous variable. **(B)** The bar plot showing standardized effects (direct, indirect, and total effect) derived from the PLS-PM.

The PLS-PM indicated that DTC, abiotic environmental variables, and bacterial community affected sediment antibiotic resistomes in an indirect manner, whereas antibiotics showed non-significant effects on antibiotic resistomes (Figure 5). DTC indirectly impacted the profile of ARGs by significantly affecting environmental variables (λ = 0.515, *p* < 0.01), bacterial community (λ = 0.375, *p* < 0.05), and MGEs (λ = −0.439, *p* < 0.05). Abiotic environmental conditions (λ = 0.388, *p* < 0.05) and bacterial community composition (λ = 0.492, *p* < 0.01) indirectly shaped the ARG profile by strongly affecting MGEs (λ = 0.656, *p* < 0.001). Thus, MGEs as a whole was identified as a direct determinant of sediment antibiotic resistomes.

## Discussion

### Terrestrial disturbance strength and natural factors largely shape the geographic pattern of sediment antibiotic resistomes

Environmental pollutions caused by heavy metals, organic compounds (such as polycyclic aromatic hydrocarbons, aniline, and nitrobenzene), and antibiotics can impose stress on microorganisms, which may increase the richness of antibiotic resistance genes (ARGs; Chen et al., 2013b; Lu and Lu, 2020; Lu et al., 2021). According to the ecological fitness-cost theory (Andersson and Hughes, 2010), weakening or eliminating these stresses can gradually reduce and eventually restore ARG richness to the pre-disturbance levels. Our results showed that the richness of ARGs in sediments decreased from the bays to offshore, and all the ARGs detected in the nearshore and offshore areas could also be detected in the bays, suggesting a declining trend of ARG richness with the decrease in anthropogenic disturbance intensity. Previous studies have shown that the concentrations of heavy metals (Chen et al., 2013b; Lu and Lu, 2020), nutrients (Zhao et al., 2017), and antibiotics (Lu et al., 2021) were positively correlated with the ARG richness in coastal sediments, indicating that ARG richness may decrease as the concentration of these contaminants reduced. However, in our study, no significant correlations were observed between the concentrations of heavy metals (except As) or nutrients and ARG richness. We also found ARG richness was strongly correlated with water depth and sediment texture (the proportions of slit and sand; Supplementary Table S1), which were mainly shaped by local hydrological and geological conditions (Black et al., 1996; Liang et al., 2020), suggesting that the accumulation of ARG richness is largely related to local natural environment. The richness of ARGs detected in this study varied considerably between zones. For example, in the bay areas, ARG richness in HZ was much lower than that in XS or SM, which may be attributed to differences in environmental conditions and anthropogenic disturbances among these three bays (Yang et al., 2011; Chen et al., 2015; Su et al., 2020a). Moreover, Chen et al. demonstrated a substantial heterogeneity of ARG profiles in the sediments of HZ (Chen J. et al., 2020), which may be associated with distance dilution in coastal area (Lu et al., 2021). Additionally, the dual action of the extensive input of freshwater from the Qiantang River and the associated ebb and flow (Wang et al., 2015) intensify the dilution of ARGs in the sediments over distance. These also partly explained that the richness of ARGs in HZ was lower than that of XS or SM.

Studies on different river basins have identified a strong positive correlation between anthropogenic disturbance intensity and normalized abundance of ARGs (Guo et al., 2018; Zheng et al., 2018; Peng et al., 2020). We found that the normalized abundance of total detected ARGs decreased as the distance to the coastline (DTC) increased, indicating a decrease in the abundance of ARGs with the decline of anthropogenic disturbance intensity from bay, nearshore, to offshore areas. On the other hand, natural factors may also contribute to the gradient of ARG abundance from the bays to offshore area. For example, we found strong positive correlations between the proportion of clay and slit (particle-size <20 μm) and the total abundance of ARGs, corresponding to a higher abundance of ARGs detected in sediment particles with size <75 μm than those with greater size (Zuo et al., 2022). This is probably due to the enhanced adsorption of microorganisms and extracellular DNA (including ARGs) onto the particles with smaller size (Jackson and Weeks, 2008; Czekalski et al., 2014).

We found antibiotic resistomes fit a classic distance-decay biogeographic pattern, suggesting provinciality of them. For example, the unique composition of ARGs in HZ could be associated with relatively high concentrations of NH_4_^+^-N and NO_3_^−^-N there. Correspondingly, some previous studies demonstrated that nitrogen level largely shaped the distribution of ARGs in soils (Forsberg et al., 2014), marine sediments (Guo et al., 2016), and rivers (Zheng et al., 2017). We detected *qacEΔ1* gene, associated with resistance to quaternary ammonium salts, in high abundance and as an indicator ARG in HZ. A large-scale pig farm (10,000 animals per year) is located in the northern HZ for 30 years, and the discharges from this pig farm have led to a significant increase of ARGs in the surrounding soil (Zhu et al., 2013) and high abundance of *qacEΔ1* gene (Johnson et al., 2016) was also detected there. Additionally, a large amount of sewage treatment plants (Su et al., 2020b) and intensive estuarine aquaculture (Yuan et al., 2019) around HZ contributed a lot to ARG spreading and accumulation in coastal sediments there, due to the low removal efficiency of ARGs by current sewage treatment procedures and the overuse of antibiotics in aquaculture. Therefore, the livestock industry, aquaculture, and sewage treatment plants around HZ were likely essential sources of ARGs of the surrounding sediments. Moreover, XS and SM, serving as two major aquaculture bases in the East China Sea, have been committed to developing mariculture for 40 years (Yang et al., 2011; Chen et al., 2015). XS possesses large mariculture area accounting for approximately one sixth of total bay area, thus being subjected to a considerable influx of antibiotics from mariculture (Gao et al., 2012; Su et al., 2020a). Meanwhile, mariculture has already resulted in a substantial influx of organic and inorganic pollutants in SM (Liang et al., 2021; Yao et al., 2022). The impact of maricultural activities on coastal antibiotic resistomes has been observed across bay area (Zhao et al., 2017), China’s coastline (Gao et al., 2018), and Northern Baltic Sea (Muziasari et al., 2016). Correspondingly, some indicator ARGs of XS and SM, including *blaPAO, ermA*, *floR, tetT,* and *strB*, were frequently detected in fishmeal (Han et al., 2017) and sediments in the mariculture zone (Muziasari et al., 2016) in high abundance, indicating maricultural sources as typical anthropogenic emissions that affect sediment antibiotic resistomes in these two bays. Collectively, natural factors and terrestrial disturbances simultaneously shape geographic patterns of sediment antibiotic resistomes in coastal northern Zhejiang.

In this study, the normalized abundance of different types of ARGs showed inconsistency in their decreasing rate with DTC. Although most ARGs tended to dilute over distance, the transmission pathway may differ between different types of ARGs. It is worth to note that the abundance of vancomycin resistance genes did not significantly decrease from the bay to the offshore area, suggesting they may possess higher risk of spread. Factually, a clear increasing trend of vancomycin resistance genes with latitude were observed in estuarine and coastal environments on the global scale (Zheng et al., 2021). Since vancomycin antibiotics are regarded as the last line of defense against pathogenic bacteria (Gilmore and Hoch, 1999), their long-distance transmission processes in marine benthic environment and potential risks need further evaluation. A limit of this study is that the absolute abundances of ARGs per unit sediment were not quantified due to the depletion of samples, and thus the contamination level or intensity of ARGs in the sediments across the ecological gradients could not be evaluated. Given that ARGs have been considered as emerging contaminants (Pruden et al., 2006), understanding their contamination intensity is crucial to comprehensively assess their ecological risks. Moreover, microbial biomass in the benthic environment may vary with different environmental conditions (Tao et al., 2017), thus influencing the robustness of characterizing resistomes in terms of the relative abundance of ARGs. Therefore, future efforts should be made to reveal resistomes based on the absolute abundance of ARGs.

### Dissemination of antibiotic resistomes across ecological gradients

Although many previous studies have considered the level of antibiotics as a major determinant of the abundance of ARGs (Xu et al., 2015; Pan and Chu, 2018; Zhang et al., 2018), the relationship between the level of different types of antibiotics and the abundance of corresponding ARGs showed inconsistent patterns. Specifically, positive, negative, or even non-significant correlations between antibiotics and corresponding ARGs were reported in various studies (Gao et al., 2012; Rodriguez-Mozaz et al., 2015; Zhu et al., 2017; Zhao et al., 2021). Here, we found that the level of antibiotics showed no associations with either relative abundance of corresponding ARGs or composition of antibiotic resistomes. This may attribute to the differences in environmental behaviors of antibiotics and spread mechanisms of ARGs in the sediment, which are likely restrict the emergence of consistent patterns.

Common associations between ARGs and MGEs were found, suggesting that MGEs could play a central role in the dissemination of ARGs across the sediments, which is also supported by the results of Partial Least Squares Path Modeling (PLS-PM). MGEs (including integrons and transposons) mediate horizontal gene transfer, which is a dominant way for the extensive dissemination of ARGs (Cambray et al., 2010; Stalder et al., 2012). We found integrase genes (*intI-1* and *cIntI-1*) showing significant positive correlations with almost all ARG types (except aminoglycoside resistance genes), and the network further illustrated the specific ARGs strongly correlated with *intI-1* and *cIntI-1*, suggesting that these genes were likely incorporated in integrons. Additionally, no significant correlations were found between transposase genes and ARGs here, which is consistent with previous reports in soil amended with sewage sludge (Chen et al., 2016), riverine water (Ouyang et al., 2015), and estuarine sediments (Zhu et al., 2017). Therefore, we speculate that integrons rather than transposons are crucial in the spread of sediment ARGs in the study region. Given the limits that only two integrase genes (*intI-1* and *cIntI-1*) were targeted here, and most of integrases do not belong to clinical classes and are diverse in the marine environment (Abella et al., 2015), more comprehensive profiles of integrase genes should be involved to test the speculation in future investigations.

On the other hand, previous studies have found that vertical transfer is also an important pathway for the dissemination of ARGs in soils (Forsberg et al., 2014) and sewage sludge (Su et al., 2015). We also found that antibiotic resistome significantly correlated with bacterial community composition, and the latter directly associated with MGEs as suggested by the PLS-PM. Thus, MGEs determined by bacterial community composition were likely the major determinant driving the dissemination of ARGs in the sediments across environment gradients. Although 8 MGEs were targeted here, no specific plasmid primers were designed, thus lacking sufficient evidence to delineate the horizontal transfer of ARGs. Although several resistance islands, including various ARGs embedded in MGEs, were confirmed as a horizontal gene transfer channel in swine agriculture (Johnson et al., 2016), further studies are needed to provide direct evidence for the hypothesis that horizontal gene transfer exacerbated the dissemination of ARGs across the sediments.

Despite the broad recognition of anthropogenic activities shaped antibiotics resistomes in coastal environments (Zhu et al., 2017; Zhang Y. et al., 2018, Zhang et al., 2020; Lu et al., 2021), our results furtherly highlight that the interplay of natural factors and terrestrial disturbance largely shape sediment antibiotic resistomes *via* affecting horizontal transfer of genes. Given that the distribution pattern of ARGs in coastal sediments could serve as indicators of terrestrial disturbance intensity, long-term monitoring should be conducted to further understand the dissemination processes and assembly mechanisms of antibiotic resistomes and their ecological risk in benthic ecosystems.

## Conclusion

This study systematically investigated the geographic patterns and key determinants of coastal sediment antibiotic resistomes across complex ecological gradients at a regional scale (some of which indicated a gradient of anthropogenic disturbance strength). The geographic patterns of antibiotic resistome suggest a strong influence of terrestrial disturbances (including anthropogenic activities like mariculture) on the occurrence and dissemination of ARGs. Specifically, terrestrial disturbance strength directly affected abiotic environmental conditions and bacterial community composition, and indirectly affected antibiotic resistome *via* MGEs that largely determined by bacterial community. Our results provide insights into regional variability of sediment antibiotic resistomes and its shaping path across complex ecological gradients, highlighting terrestrial disturbances as major determinative forces in shaping antibiotic resistomes. This work has positive implications for establishing strategies to mitigate the spread of ARGs from terrestrial runoffs and anthropogenic activities to the ocean.

## Data availability statement

The datasets presented in this study can be found in online repositories. The names of the repository/repositories and accession number(s) can be found in the article/Supplementary material.

## Author contributions

DZ and KW designed the study. SX, ML, KW, and DZ performed the study. KW, SX, and DZ proposed the data analysis strategy. SX, HY, and YW analyzed the data. HY and DH assisted with the analytic tools. SX and KW wrote the manuscript. All authors contributed to the article and approved the submitted version.

## Funding

This work was funded by the National Key Research and Development Program of China (2016YFC1402205), National Natural Science Foundation of China (41977192), Natural Science Foundation of Ningbo (2021 J060 and 2017A610302), Fundamental Research Funds for the Provincial Universities of Zhejiang (SJLY2022001), Zhejiang Provincial Top Discipline of Biological Engineering (ZS2012006), and K.C. Wong Magna Fund in Ningbo University.

## Conflict of interest

The authors declare that the research was conducted in the absence of any commercial or financial relationships that could be construed as a potential conflict of interest.

## Publisher’s note

All claims expressed in this article are solely those of the authors and do not necessarily represent those of their affiliated organizations, or those of the publisher, the editors and the reviewers. Any product that may be evaluated in this article, or claim that may be made by its manufacturer, is not guaranteed or endorsed by the publisher.

## Footnotes

^1^ https://www.arb-silva.de/
