# Supplementary material for: Geographic patterns and determinants of antibiotic resistomes in coastal sediments across complex ecological gradients
Source: Front Microbiol. 2022 Nov 3;13:922580. doi: 10.3389/fmicb.2022.922580 (PMC9669582; doi:10.3389/fmicb.2022.922580)

Supplementary Material

1 Supplementary Tables

Table S1 Pearson correlations of environmental variables (normalized except pH) with distances to coastline (DTC; log_{10} transformed) and the richness and normalized abundance of antibiotic resistance genes (ARGs).

Environmental variables	DTC	Richness of ARGs	Normalized abundance of ARGs
Clay	**-0.489***	0.319	**0.575****
Slit	**-0.433***	**0.448****	**0.679****
Sand	**0.447***	**-0.423***	**-0.673****
pH	**0.517****	0.137	0.132
TC	**0.433***	-0.061	-0.249
TN	-0.130	0.180	0.118
TS	-0.262	0.092	0.125
NH_4^+-N	**-0.412***	-0.037	-0.017
NO_3^--N	**-0.343***	0.071	0.191
TP	**-0.424***	0.073	0.195
As	**-0.373***	**0.407****	**0.446****
Cd	0.079	0.238	0.145
Cr	0.060	-0.080	0.041
Cu	**-0.407***	0.339*	**0.374***
Hg	-0.113	0.099	0.233
Ni	**-0.457****	0.273	**0.404***
Pb	-0.143	-0.078	0.256
Zn	**-0.453****	0.109	**0.318***
Depth	0.060	**-0.397***	**-0.389***

Data present correlation coefficients (r; n=32), and bold values indicate significant correlations; $^{*}p < 0.05$, $^{**}p < 0.01$. TN, total nitrogen; TC, total carbon; TS, total sulfur; TP, total phosphorus; Depth, water depth.

Table S2 The unique and shared antibiotic resistance genes (ARGs) in the bay, nearshore, and offshore samples.

Gene	ARG type	Location detected	Abundance*
aac(6')I1	Aminoglycoside	Bays	57
aac(6')-II	Aminoglycoside	Bays	54
aac(6')-lb(akaaacA4)-01	Aminoglycoside	Bays	513
aacC1	Aminoglycoside	Bays	331
aadA-01	Aminoglycoside	Bays	154
aadA2-01	Aminoglycoside	Bays	52
aadA9-02	Aminoglycoside	Bays	106
spcN-02	Aminoglycoside	Bays	10
blaCTX-M-03	Beta-Lactamase	Bays	226
blaCTX-M-05	Beta-Lactamase	Bays	503
blaOXA10-01	Beta-Lactamase	Bays	217
blaOXA10-02	Beta-Lactamase	Bays	229
mecA	Beta-Lactamase	Bays	20
tetA-02	Tetracycline	Bays	5
tetE	Tetracycline	Bays	7
tetM-02	Tetracycline	Bays	12
tetO-01	Tetracycline	Bays	8
tetPB-01	Tetracycline	Bays	6
tetX	Tetracycline	Bays	13
mphB	MLSB	Bays	2
pikR1	MLSB	Bays	4
ermF	MLSB	Bays	7
cfr	FCA	Bays	6
vanYB	Vancomycin	Bays	3
fabK	Multidrug	Bays	2
ereB	Multidrug	Bays	7
bacA-01	Multidrug	Bays	11
blaROB	Beta-Lactamase	Nearshore	6
tetR-03	Tetracycline	Nearshore	16
vanC-01	Vancomycin	Nearshore	4
qacA	Multidrug	Nearshore	5
tetPA	Tetracycline	Offshore	7
aadA2-02	Aminoglycoside	Bays+Nearshore	29
aadA5-01	Aminoglycoside	Bays+Nearshore	14
aadE	Aminoglycoside	Bays+Nearshore	18
tetM-01	Tetracycline	Bays+Nearshore	14
ereA	MLSB	Bays+Nearshore	55
ermX	MLSB	Bays+Nearshore	16
carB	MLSB	Bays+Nearshore	5
yidY_mdtL-02	FCA	Bays+Nearshore	31
catB3	FCA	Bays+Nearshore	65
sul2	Sulfonamide	Bays+Nearshore	68
vanRD	Vancomycin	Bays+Nearshore	6
acrR-02	Multidrug	Bays+Nearshore	35

Gene	ARG type	Location detected	Abundance*
bacA-02	Multidrug	Bays+Nearshore	24
qacEΔ1-01	Multidrug	Bays+Nearshore	2724
qacH-02	Multidrug	Bays+Nearshore	50
rarD-02	Multidrug	Bays+Nearshore	19
yceE_mdtG-01	Multidrug	Bays+Nearshore	44
yceL_mdtH-02	Multidrug	Bays+Nearshore	27
bla-AAC-1	Beta-Lactamase	Bays+Offshore	12
lnuC	MLSB	Bays+Offshore	14
tetL-02	Tetracycline	Bays+Offshore	54
tetV	Tetracycline	Bays+Offshore	20
vanHD	Vancomycin	Bays+Offshore	18
aac	Aminoglycoside	Bays+Nearshore+ Offshore	65
aac(6')-lb(akaaacA4)-02	Aminoglycoside	Bays+Nearshore+ Offshore	171
aac(6')-lb(akaaacA4)-03	Aminoglycoside	Bays+Nearshore+ Offshore	59
aacC	Aminoglycoside	Bays+Nearshore+ Offshore	3370
aacC4	Aminoglycoside	Bays+Nearshore+ Offshore	95
aadA-02	Aminoglycoside	Bays+Nearshore+ Offshore	172
aadA1	Aminoglycoside	Bays+Nearshore+ Offshore	238
aadA2-03	Aminoglycoside	Bays+Nearshore+ Offshore	180
aadA5-02	Aminoglycoside	Bays+Nearshore+ Offshore	201
aadA9-01	Aminoglycoside	Bays+Nearshore+ Offshore	52
aphA1(aka_kanR)	Aminoglycoside	Bays+Nearshore+ Offshore	6471
strB	Aminoglycoside	Bays+Nearshore+ Offshore	948
ampC-01	Beta-Lactamase	Bays+Nearshore+ Offshore	196
ampC-02	Beta-Lactamase	Bays+Nearshore+ Offshore	290
ampC-04	Beta-Lactamase	Bays+Nearshore+ Offshore	592
ampC-07	Beta-Lactamase	Bays+Nearshore+ Offshore	8
ampC-09	Beta-Lactamase	Bays+Nearshore+ Offshore	46

Gene	ARG type	Location detected	Abundance*
blaCMY2-02	Beta-Lactamase	Bays+Nearshore+ Offshore	339
blaCTX-M-01	Beta-Lactamase	Bays+Nearshore+ Offshore	322
blaCTX-M-02	Beta-Lactamase	Bays+Nearshore+ Offshore	91
blaCTX-M-04	Beta-Lactamase	Bays+Nearshore+ Offshore	1021
blaIMP-01	Beta-Lactamase	Bays+Nearshore+ Offshore	41
bla-L1	Beta-Lactamase	Bays+Nearshore+ Offshore	41
blaOXY	Beta-Lactamase	Bays+Nearshore+ Offshore	939
blaSFO	Beta-Lactamase	Bays+Nearshore+ Offshore	4137
blaSHV-01	Beta-Lactamase	Bays+Nearshore+ Offshore	98
blaTEM	Beta-Lactamase	Bays+Nearshore+ Offshore	3313
blaVIM	Beta-Lactamase	Bays+Nearshore+ Offshore	237
cepA	Beta-Lactamase	Bays+Nearshore+ Offshore	12
cfiA	Beta-Lactamase	Bays+Nearshore+ Offshore	9
cphA-01	Beta-Lactamase	Bays+Nearshore+ Offshore	1068
cphA-02	Beta-Lactamase	Bays+Nearshore+ Offshore	253
fox5	Beta-Lactamase	Bays+Nearshore+ Offshore	6708
penA	Beta-Lactamase	Bays+Nearshore+ Offshore	91
tetD-02	Tetracycline	Bays+Nearshore+ Offshore	299
tetG-01	Tetracycline	Bays+Nearshore+ Offshore	1183
tetG-02	Tetracycline	Bays+Nearshore+ Offshore	324
tetPB-02	Tetracycline	Bays+Nearshore+ Offshore	573
tetR-02	Tetracycline	Bays+Nearshore+ Offshore	999
mphA-01	MLSB	Bays+Nearshore+ Offshore	2055
mphA-02	MLSB	Bays+Nearshore+ Offshore	1240

Gene	ARG type	Location detected	Abundance*
matA_mel	MLSB	Bays+Nearshore+ Offshore	85
erm(34)	MLSB	Bays+Nearshore+ Offshore	27
erm(36)	MLSB	Bays+Nearshore+ Offshore	775
ermK-01	MLSB	Bays+Nearshore+ Offshore	30
oleC	MLSB	Bays+Nearshore+ Offshore	886
pikR2	MLSB	Bays+Nearshore+ Offshore	1188
vatE-01	MLSB	Bays+Nearshore+ Offshore	94
vgb-01	MLSB	Bays+Nearshore+ Offshore	93
acrA-01	FCA	Bays+Nearshore+ Offshore	93
acrA-02	FCA	Bays+Nearshore+ Offshore	73
acrA-03	FCA	Bays+Nearshore+ Offshore	41
acrA-04	FCA	Bays+Nearshore+ Offshore	1276
acrA-05	FCA	Bays+Nearshore+ Offshore	2860
acrB-01	FCA	Bays+Nearshore+ Offshore	27
acrF	FCA	Bays+Nearshore+ Offshore	146
adeA	FCA	Bays+Nearshore+ Offshore	8
cmlA1-01	FCA	Bays+Nearshore+ Offshore	53
cmlA1-02	FCA	Bays+Nearshore+ Offshore	46
cmx(A)	FCA	Bays+Nearshore+ Offshore	79
floR	FCA	Bays+Nearshore+ Offshore	398
mexA	FCA	Bays+Nearshore+ Offshore	652
mexE	FCA	Bays+Nearshore+ Offshore	108
mexF	FCA	Bays+Nearshore+ Offshore	65194

Gene	ARG type	Location detected	Abundance*
oprJ	FCA	Bays+Nearshore+ Offshore	9140
yidY_mdtL-01	FCA	Bays+Nearshore+ Offshore	115
vanB-01	Vancomycin	Bays+Nearshore+ Offshore	618
vanC-03	Vancomycin	Bays+Nearshore+ Offshore	2313
vanC2_vanC3	Vancomycin	Bays+Nearshore+ Offshore	64
vanHB	Vancomycin	Bays+Nearshore+ Offshore	401
vanRB	Vancomycin	Bays+Nearshore+ Offshore	87
vanSB	Vancomycin	Bays+Nearshore+ Offshore	140
vanTC-02	Vancomycin	Bays+Nearshore+ Offshore	68
vanXD	Vancomycin	Bays+Nearshore+ Offshore	50
vanYD-01	Vancomycin	Bays+Nearshore+ Offshore	363
ceoA	Multidrug	Bays+Nearshore+ Offshore	724
emrD	Multidrug	Bays+Nearshore+ Offshore	456
marR-01	Multidrug	Bays+Nearshore+ Offshore	105
mdtE_yhiU	Multidrug	Bays+Nearshore+ Offshore	187
mepA	Multidrug	Bays+Nearshore+ Offshore	13
acrR-01	Multidrug	Bays+Nearshore+ Offshore	688
mtrC-01	Multidrug	Bays+Nearshore+ Offshore	162
mtrC-02	Multidrug	Bays+Nearshore+ Offshore	63
mtrD-02	Multidrug	Bays+Nearshore+ Offshore	288
mtrD-03	Multidrug	Bays+Nearshore+ Offshore	114
oprD	Multidrug	Bays+Nearshore+ Offshore	3361
pncA	Multidrug	Bays+Nearshore+ Offshore	2970
qacEΔ1-02	Multidrug	Bays+Nearshore+ Offshore	2385

Gene	ARG type	Location detected	Abundance*
qacH-01	Multidrug	Bays+Nearshore+ Offshore	703
tolC-02	Multidrug	Bays+Nearshore+ Offshore	32
tolC-03	Multidrug	Bays+Nearshore+ Offshore	181
ttgA	Multidrug	Bays+Nearshore+ Offshore	27
ttgB	Multidrug	Bays+Nearshore+ Offshore	93
yceL_mdtH-01	Multidrug	Bays+Nearshore+ Offshore	508
yceL_mdtH-03	Multidrug	Bays+Nearshore+ Offshore	93

Abundance*, average normalized abundance in unit of $\times 10^{-6}$ copies per cell. FCA, fluoroquinolone, quinolone, florfenicol, chloramphenicol and amphenicol; MLSB, Macrolide-Lincosamide-Streptogramin B.

Table S3 Analysis of Similarity (ANOSIM) based on Bray-Curtis dissimilarity for testing pairwise difference in the composition of antibiotic resistomes between zones (Global R = 0.486, $p < 0.01$, 9999 permutations).

	HZ	XS	SM	ZS	JICR	IC
XS	**0.604***					
SM	**0.748****	**0.276****				
ZS	**0.613****	**0.500****	**0.588****			
JICR	**0.637****	**0.438***	**0.344***	0.020		
IC	**0.538***	**0.620***	**0.772****	**0.438****	**0.531****	
YS	**0.594***	**0.444****	**0.575****	**0.594****	**0.458***	**0.525****

Bold R values present significant differences; $^{*}p < 0.05$, $^{**}p < 0.01$. HZ, Hangzhou Bay; XS, Xiangshan Bay; SM, Sanmen Bay; ZS, Zhoushan Islands; JICR, Jiushan Islands Conservation and Reservation; IC, the Eastern Boundary of the Island-Chain; YS, Yushan Islands Reservation.

Table S4 Indicator antibiotic resistance genes (ARGs) of each zone.

Zone	Indicator ARGs	Test-statistic	*p*
HZ	*qacEΔ1-01*	0.887	0.001
	qacEΔ1-02	0.773	0.020
	acrR-01	0.766	0.004
	qacH-01	0.757	0.003
	aadA1	0.747	0.007
	aadA-01	0.717	0.014
XS	*tetT*	0.760	0.003
	tetX	0.725	0.002
	floR	0.718	0.014
	strB	0.688	0.002
SM	*ermA*	0.927	0.001
	blaPAO	0.829	0.004
	bla-AAC-1	0.803	0.006
	mecA	0.754	0.006
	sul2	0.735	0.006
YS	*vanC2_vanC3*	0.851	0.002
	ermK-01	0.609	0.037
IC	*ttgB*	0.701	0.003

HZ, Hangzhou Bay; XS, Xiangshan Bay; SM, Sanmen Bay; IC, the Eastern Boundary of the Island-Chain; YS, Yushan Islands Reservation.

Table S5 Pearson correlation of the normalized abundance of different types of antibiotic resistance genes (ARGs) with that of integrons (*cIntI-1 and intI-1*) and transposons (*tnpA-02, tnpA-04, tnpA-05, and Tp614*). The types of ARGs were classified based on the antibiotic to which they confer resistance.

	cIntI-1	***intI-1***	**All Integrons**	***tnpA-02***	***tnpA-04***	***tnpA-05***	***Tp614***	**All Transposase**	**All MGEs**
**Aminoglycoside**	0.269	0.227	0.253	0.190	-0.087	-0.029	0.074	-0.084	0.222
**Beta-Lactamase**	**0.799****	**0.859****	**0.859****	**0.803****	-0.164	-0.164	0.051	-0.149	**0.789****
**FCA**	**0.864****	**0.845****	**0.881****	**0.813****	0.008	-0.123	0.017	0.024	**0.856****
**MLSB**	**0.898****	**0.957****	**0.960****	**0.939****	-0.186	-0.197	0.169	-0.168	**0.881****
**Multidrug**	**0.714****	**0.852****	**0.816****	**0.727****	0.033	0.154	0.013	0.049	**0.800****
**Sulfonamide**	**0.539***	**0.603****	**0.592****	**0.578****	-0.139	-0.133	-0.004	-0.128	**0.537***
**Tetracycline**	**0.902****	**0.743****	**0.839****	**0.753****	0.029	-0.024	0.144	0.044	**0.822****
**Vancomycin**	**0.871****	**0.974****	**0.958****	**0.915****	-0.135	-0.159	0.072	-0.118	**0.893****
**Multidrug**	**0.796****	**0.552****	**0.681****	**0.587****	0.122	0.130	0.199	0.134	**0.694****
**All ARGs**	**0.817****	**0.893****	**0.886****	**0.801****	-0.050	0.022	0.063	-0.034	**0.846****

Data present correlation coefficient (r; n=32), and bold values indicate significant correlations; $^{*}p < 0.05$; $^{**}p < 0.01$. FCA, fluoroquinolone, quinolone, florfenicol, chloramphenicol and amphenicol; MLSB, Macrolide-Lincosamide-Streptogramin B.

2 Supplementary Figures

Figure S1 Geographic distribution of the concentration of antibiotics across the study region. **Tetracyclines** (methacycline (MT)); **Sulfonamides** (sulfachinoxalin (SCX)); **Fluoroquinolones** (including fleoxacin (FL), ofloxacin (OFX)); marbofloxacin (MAR), pefloxacin (PEF), difluoxacin (DIF), and lomefloxacin (LFX)); **Macrolides** (including erythromycin (ERM), clarithromycin (CTM), and roxithromycin (RTM)); **Chloramphenicols** (chloramphenicol (CAP)).

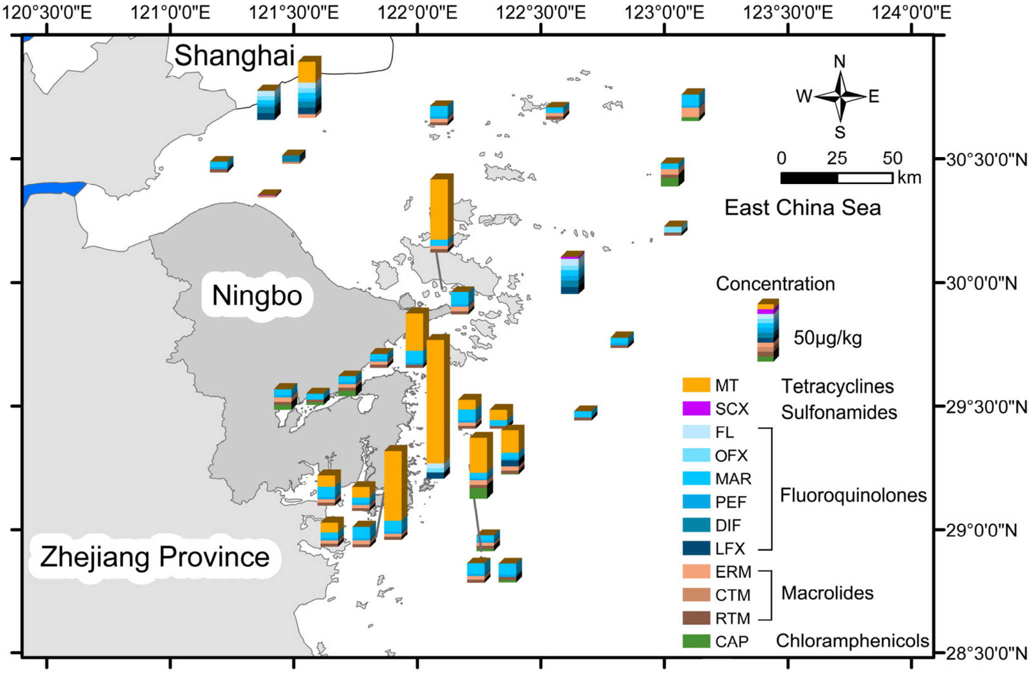

Figure S2 Kriged maps showing spatial distribution of major sediment environmental variables across the study region. (A) Clay, (B) Slit, (C) Sand, (D) pH, (E) Total Carbon, (F) Total Nitrogen, (G) Total Sulfur, (H) Ammonium, (I) Nitrate, (J) Total Phosphorus, (K) Arsenic, (L) Cadmium, (M) Chromium, (N) Copper, (O) Mercury, (P) Nickel, (Q) Lead, and (R) Zinc.

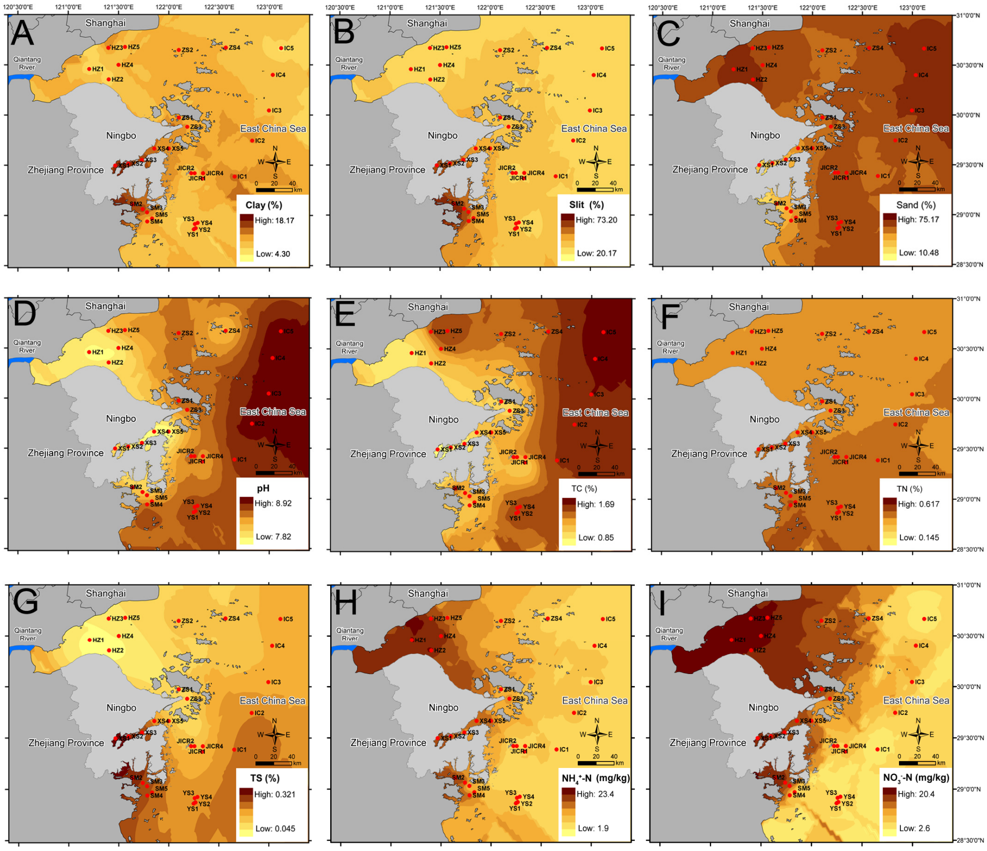

Figure S2 Continued.

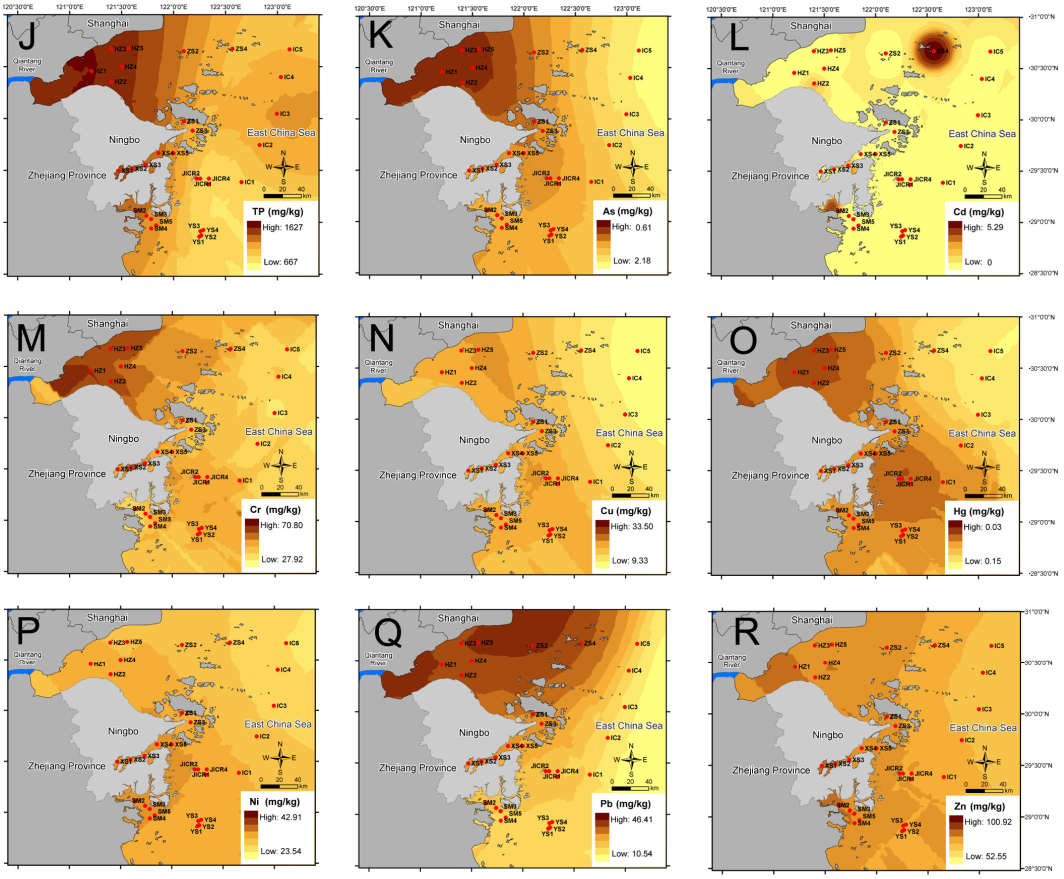

Figure S3 Principal Coordinate Analysis (PCoA) based Bray-Curtis dissimilarity showing compositional variation of sediment bacterial communities across zones of coastal northern Zhejiang (A). A linear fit showing the correlation between PCoA1 value and distance to coastline with adjusted R^2 (B). The shaded area represents 95% confidence intervals.

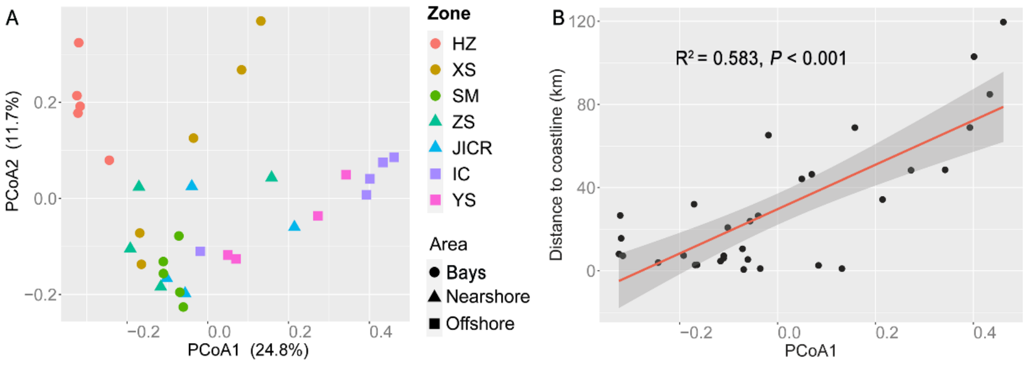

Figure S4 The correlations between the normalized abundance of different types of antibiotic resistance genes (ARGs) and distance to coastline (DTC). The red line represents a linear fit. The shaded area represents 95% confidence intervals. All R^2 values were adjusted. FCA, fluoroquinolone, quinolone, florfenicol, chloramphenicol and amphenicol; MLSB, macrolide-lincosamide-streptogramin B.

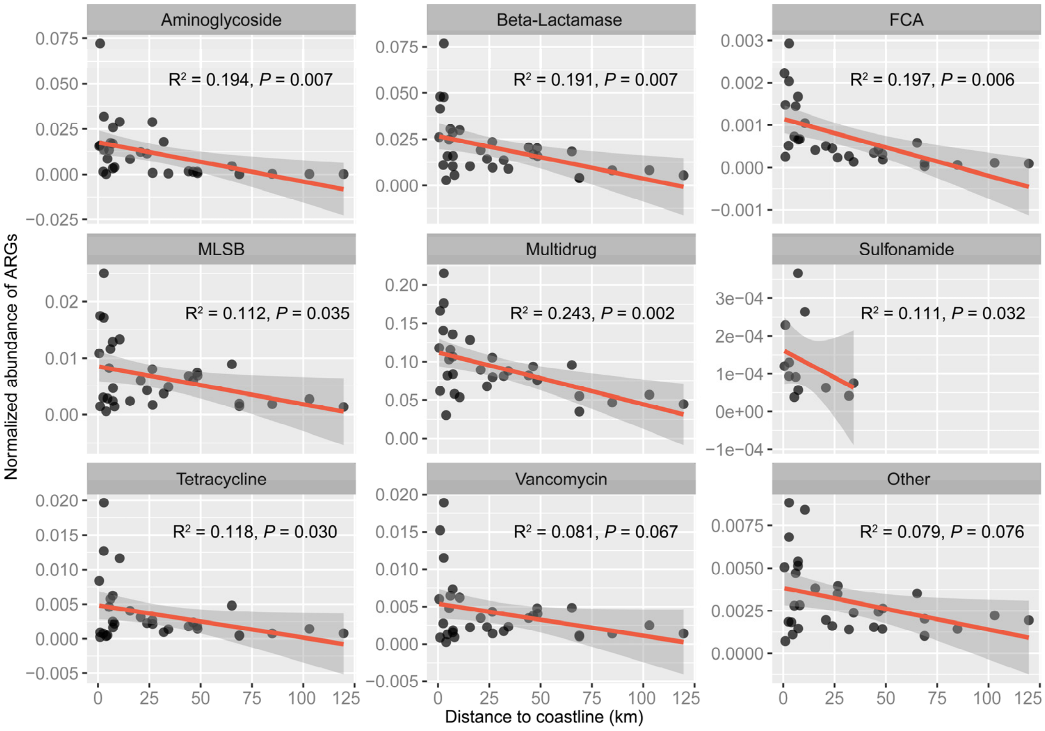

Figure S5 Histogram maps showing spatial distribution of the normalized abundance of mobile genetic elements (MGEs) across the study region.

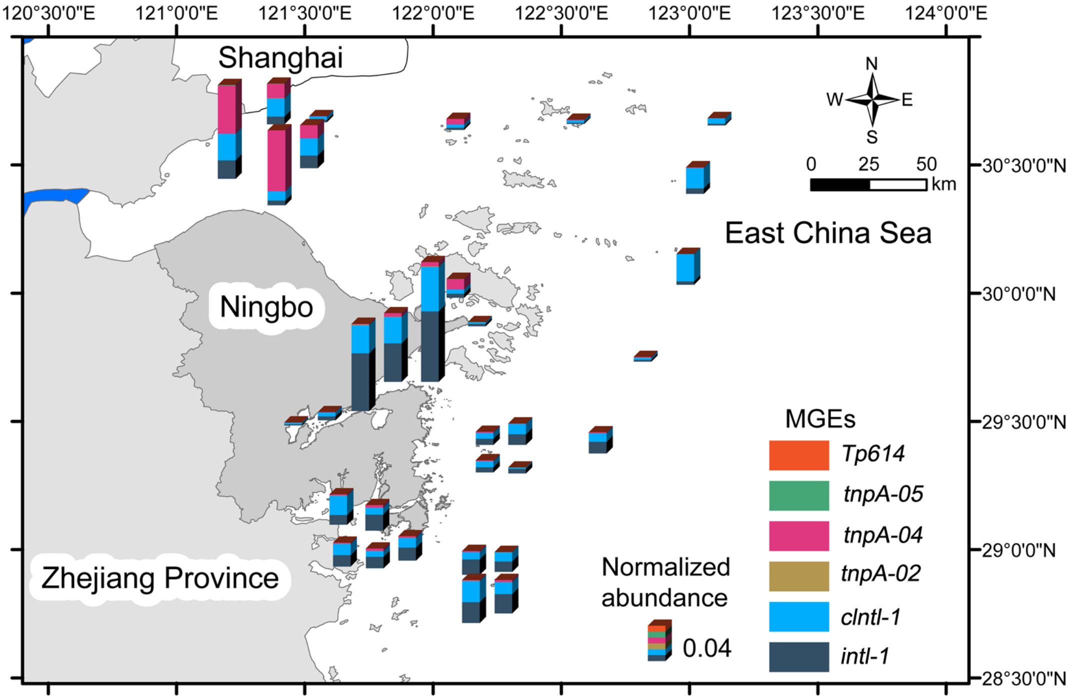

Figure S6 Characteristics of mobile genetic elements (MGEs) across bay, nearshore, and offshore areas. Boxplots showing the variation in normalized abundance of MGEs (A). Statistical significance was tested by Wilcoxon rank-sum test ($^{*}p < 0.05$, $^{**}p < 0.01$, ns: not significant). The lower and upper hinges of the boxes correspond to the 25th and 75th percentiles. The lines in the boxes correspond to median. The upper whisker extends from the hinge to the value no further than 1.5IQR (inter-quartile range). The lower whisker extends from the hinge to the smallest value at most 1.5IQR. Data beyond the end of the whiskers are 'outlying' points. A linear fit showing the correlation between the normalized abundance of MGEs and distance to coastline (DTC) with adjusted R^2 (B). The shaded area represents 95% confidence intervals.

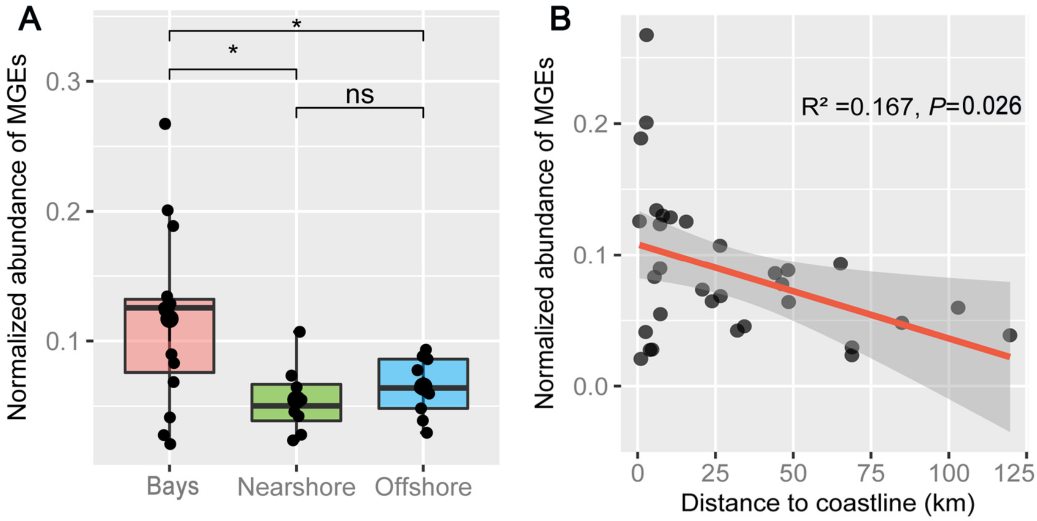

Figure S7 Redundancy analysis (RDA) showing the associations between antibiotic resistomes and environmental variables. Environmental variables were co-plotted to identify key factors that shape the antibiotic resistomes. Environmental variables were normalized to reduce the effect of unit differences. The "vif.cca" function of the R package "vegan" was used to remove redundant constraints (with variance inflation factors >10). And then the "envfit" function was used to select variables correlated with antibiotic resistomes ($p < 0.05$) for generating the final ordinations. DTC, distance to coastline; TN, total nitrogen.

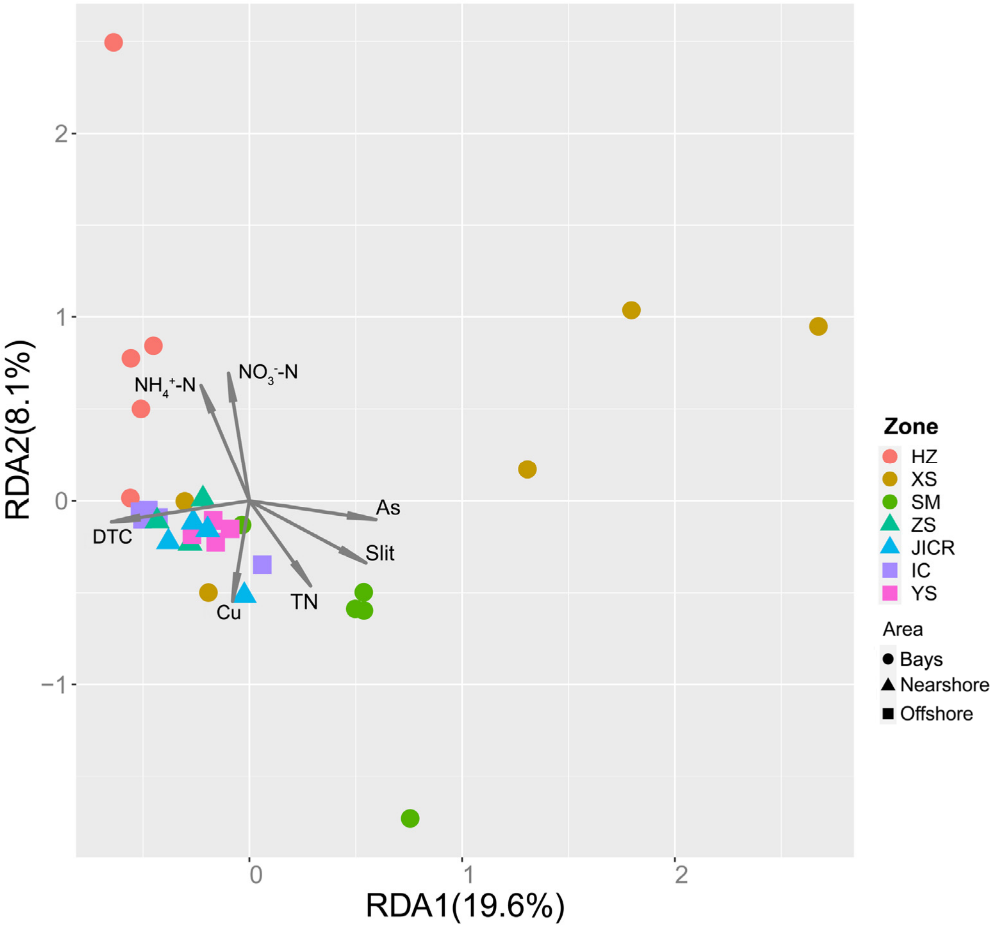

Figure S8 Testing Spearman correlations between the concentration of antibiotics and the normalized abundance of each type of antibiotic resistance genes (ARGs). Colors of the heatmap cells present correlation coefficients and data in the cells present *p*-values. FCA, fluoroquinolone, quinolone, florfenicol, chloramphenicol and amphenicol; MLSB, macrolide-lincosamide-streptogramin B.

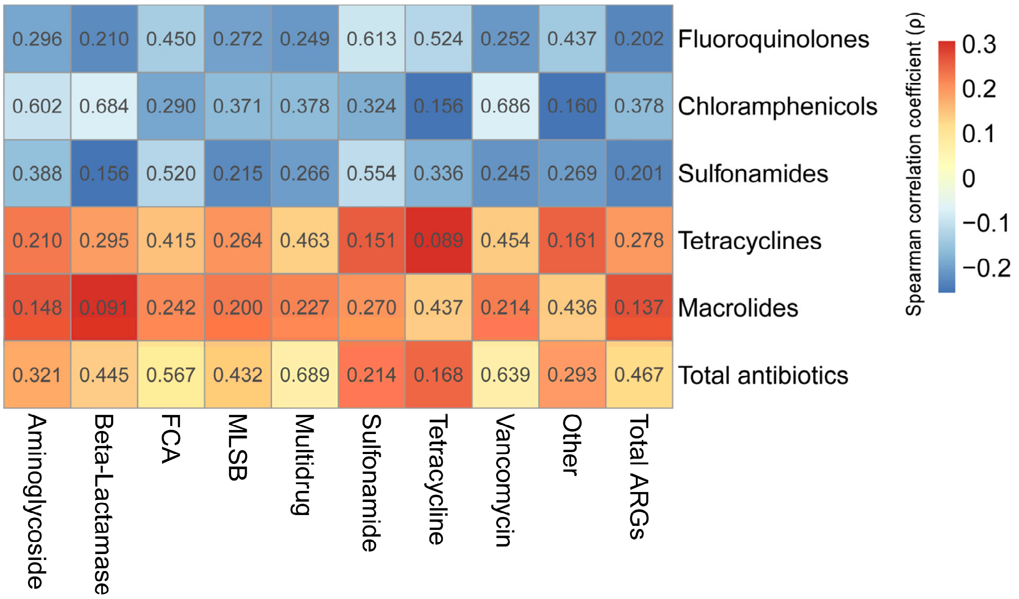

Supplement: Supplementary file 1 [file Data_Sheet_1.pdf]
